# Mammillary body injury in neonatal encephalopathy: a multicentre, retrospective study

**DOI:** 10.1038/s41390-021-01436-3

**Published:** 2021-03-02

**Authors:** Maarten. H. Lequin, Sylke. J. Steggerda, Mariasavina Severino, Domenico Tortora, Alessandro Parodi, Luca A. Ramenghi, Floris Groenendaal, Karlijn M. E. Meys, Manon J. N. L. Benders, Linda S. de Vries, Seralynne D. Vann

**Affiliations:** 1 Department of Radiology, University Medical Center Utrecht, Utrecht, The Netherlands; 2 Department of Neonatology, Leiden University Medical Center, Leiden, The Netherlands; 3 Neuroradiology Unit, IRCCS Istituto Giannina Gaslini, Genoa, Italy; 4 Neonatal Intensive Care Unit, Department Mother and Child, IRCCS Istituto Giannina Gaslini, Genoa, Italy; 5 Department of Neurosciences, Rehabilitation, Ophthalmology, Genetics, Maternal and Child Health (DINOGMI), University of Genoa, Genoa, Italy; 6 Department of Neonatology, UMC Utrecht Brain Center, Utrecht University, Utrecht, The Netherlands; 7 School of Psychology, College of Biomedical and Life Sciences, Cardiff University, Cardiff, UK

## Abstract

**Background:**

The mammillary bodies (MBs) have repeatedly been shown to be critical for memory, yet little is known about their involvement in numerous neurological conditions linked to memory impairments, including neonatal encephalopathy.

**Methods:**

We implemented a multicentre retrospective study, assessing magnetic resonance scans of 219 infants with neonatal encephalopathy who had undergone hypothermia treatment in neonatal intensive care units located in the Netherlands and Italy.

**Results:**

Abnormal MB signal was observed in ~40% of infants scanned; in half of these cases, the brain appeared otherwise normal. MB involvement was not related to the severity of encephalopathy or the pattern/severity of hypoxic–ischaemic brain injury. Follow-up scans were available for 18 cases with abnormal MB signal; in eight of these cases, the MBs appeared severely atrophic.

**Conclusions:**

This study highlights the importance of assessing the status of the MBs in neonatal encephalopathy; this may require changes to scanning protocols to ensure that the slices are sufficiently thin to capture the MBs. Furthermore, long-term follow-up of infants with abnormal MB signal is needed to determine the effects on cognition, which may enable the use of early intervention strategies. Further research is needed to assess the role of therapeutic hypothermia in MB involvement in neonatal encephalopathy.

**Impact:**

- The MBs are particularly sensitive to hypoxia in neonates.
- Current hypothermia treatment provides incomplete protection against MB injury.
- MB involvement is likely overlooked as it can often occur when the rest of the brain appears normal.
- Given the importance of the MBs for memory, it is necessary that this region is properly assessed in neonatal encephalopathy. This may require improvements in scanning protocols.

## Introduction

The mammillary bodies (MBs) have repeatedly been shown to be important for memory, most notably from cases of Korsakoff syndrome.^1^ However, their potential involvement in other neurological conditions has been largely overlooked. We recently carried out a single-centre observational study and found preliminary evidence for MB involvement in neonatal encephalopathy;^2^ at least 13% of the 235 identified neonates had abnormal signal of the MBs. Importantly, this MB involvement was not related to the severity of encephalopathy or presence of pathology elsewhere in the brain.

For the past decade, hypothermia treatment has been used as a standard treatment for neonatal encephalopathy. Hypothermia has been shown to be effective in lessening damage to the basal ganglia, thalamus and white matter^3^ with a reduction in death and disability at 18 months.^4^ However, more recent findings have shown that despite these early improvements, school-age children who had undergone hypothermia treatment continued to have cognitive difficulties^5^ and one study reported no noticeable difference in cognitive ability when children with or without hypothermia treatment were compared.^6^ Consistent with this, our preliminary findings showed abnormal MB signal not only in infants from the period prior to hypothermia treatment but also in those infants who had undergone whole-body cooling. In fact, on the basis of this single sample, there was no evidence to suggest that this treatment approach ameliorated the effect of hypoxia on the MBs in neonates.

Given the importance of the MBs for memory processing—with recent evidence suggesting a role for this structure in coordinating hippocampal–cortical activity necessary for memory formation^7^—MB pathology resulting from neonatal encephalopathy would likely cause learning and cognitive impairments that could have long-term effects on both the child and their family. Indeed, a recent study assessing the long-term effects of neonatal encephalopathy identified hypoperfusion in structures closely connected to the MBs—including the fornix, thalamus and hippocampus—in individuals with poor long-term neurodevelopmental outcomes compared to those with good long-term outcomes.^8^ However, due to the small size of the MBs, it is likely that abnormal signal is often undetected unless the MBs are specifically examined and scanning protocols include sufficiently thin slices to capture the MBs. As such, MB involvement may be routinely missed, resulting in children not receiving suitable support and long-term follow-up assessments.

A current limitation, however, is that our preliminary findings were from a single centre, raising the possibility that this MB involvement may be atypical rather than indicative of a more widespread occurrence. The aim of the present study was to examine the replicability of these initial results by carrying out a larger study across an additional two centres, one in the Netherlands and one in Italy. Examining a greater number of cases, from different centres and countries, makes it possible to not only assess the reliability of the initial finding, that MB injury is a common finding in neonatal encephalopathy, but also to better estimate the likelihood of any occurrence.

## Methods

### Patients

A multicenter retrospective observational study was carried out, assessing brain magnetic resonance imaging (MRI) of 219 infants with neonatal encephalopathy (131 males; gestational age 35–41 weeks, median 39 weeks, interquartile range (IQR) 2 weeks). MRIs were acquired at two tertiary neonatal intensive care units: Leiden University Medical Center, The Netherlands and Gaslini Children’s Hospital in Genoa, Italy. The time period of data collection only included the era following the implementation of hypothermia treatment (Leiden, 2008–2019; Genoa, 2007–2019).

The severity grade of neonatal encephalopathy (Sarnat score I, II and III) was assessed by the neonatologist and these scores are presented along with a summary of the patient characteristics in Table 1. The study was given approval by the Institutional Review Boards at Leiden University Medical Center and Gaslini Hospital. Given the retrospective nature of the study and the use of anonymised data, a waiver of informed consent was obtained.

**Table 1:** Summary of cases with and without abnormal mammillary body (MB) signal across sites.

Centre	Number of cases with normal MB signal	Number of cases with abnormal MB signal	Grade of neonatal encephalopathy I/II/III/NA		Pattern of injury normal/watershed/BGT/NT	
			Cases with normal MB signal	Cases with abnormal MB signal	Cases with normal MB signal	Cases with abnormal MB signal
Leiden % within the MB group % of all cases in the subgroup	75	61 (44.9%)	36/24/14/1 48%/32%/19%/1% 59%/55%/48%/50%	25/20/15/1 41%/33%/24%/2% 41%/45%/52%/50%	51/6/13/5 68%/8%/17%/7% 58%/66%/59%/29%	37/3/9/12 60%/5%/15%/20% 42%/33%/41%/71%
Genoa % within the MB group % across subgroup	52	31 (37.3%)	11/34/5/2 21%/65%/10%/4% 85%/61%/42%/100%	2/22/7/0 6%/71%/23%/0% 15%/39%/58%/0%	32/10/5/5* 62%/19%/10%/10% 24%/67%/42%/33%	9/5/7/10* 29%/16%/23%/33% 22%/33%/58%/67%
Combined % within MB group % across subgroup	127	92 (41.6%)	47/58/19/3 37%/46%/15%/2% 64%/58%/46%/75%	27/42/22/1 29%/46%/24%/1% 36%/42%/54%/25%	83/16/18/10** 65%/13%/14%/8% 64%/67%/53%/31%	46/8/16/22** 50%/9%/17%/24% 36%/33%/47%/69%

NA: Could not be assessed due to high dose of sedatives. Pattern of injury: no abnormalities (normal), damage into the watershed areas, damage predominantly to the deep grey matter, that is, basal ganglia/thalamus (BGT), and severe brain damage, that is, near-total brain injury (NT).

*P = 0.007 MB abnormalities vs no MB abnormality (Genoa, *χ*^2^).

**P = 0.003 normal/watershed vs BGT/NT (Combined, *χ*^2^).

### MRI imaging acquisition and assessment

MRIs were performed within 10 days of birth (median, 5; IQR, 2; Table 2). In Leiden, scans were obtained using a 3.0 Tesla MR system (Achieva, Philips Medical Systems, Best, The Netherlands) with a neonatal head coil, using a standardised protocol. This included 3-D T1-weighted images (slice thickness 1 mm, no gap), T2-weighted images in the axial plane (slice thickness 2 mm, no gap) and diffusion-weighted sequences in the axial plane (slice thickness 4 mm, no gap, and *b* values of 0 and 1000 s/mm^2^). In Genoa, scans were obtained using 1.5 T (Intera Achieva) and 3 T (Ingenia) MRI Systems (Philips, Best, The Netherlands) with neonatal head coils and standardised protocols, including axial and coronal T2-weighted sequences (slice thickness of 2–3 mm) and axial and sagittal T1-weighted images (slice thickness of 2–4 mm). In addition, axial diffusion-weighted imaging (3–4 mm slice thickness, 0-mm section gap and *b* values of 0 and 800 s/mm^2^ (1.5 T) or *b* values of 0 and 1000 s/mm^2^ (3 T) was performed.

**Table 2:** Summary of clinical data for cases with normal and abnormal MB signal in Leiden and Genoa.

	Leiden		Genoa	
	Cases with normal MB signal (75)	Cases with abnormal MB signal (61)	Cases with normal MB signal (52)	Cases with abnormal MB signal (31)
Gestational age (median, IQR)	40.0 (3.0)**	39.0 (4.0)**	39.0 (2.0)**	39.0 (2.0)**
Day of MRI (median, IQR)	5 (1)*	6 (1)*	7.5 (5)*	6 (2)*
Male/female	41/34	32/29	38/14	20/11
Mortality (%)	14 (21.3)***	15 (24.6)***	0 (0)***	3 (8.6)***
Perinatal sentinel event (%)	17 (23)	21 (34)	5 (10)^	12 (39)^^^
Cord pH (mean, SD)	6.94 (0.20)	6.94 (0.18)	6.95 (0.14)^^^^	6.87 (0.16)^^^^
APGAR score 5 min (mean, SD)	2.71 (1.53)	3.18 (2.17)	5.40 (1.97)^^^^	4.10 (2.63)^^^^

*GA* gestational age (weeks).

*P < 0.001 Leiden vs Genoa (all cases, ANOVA).

**P = 0.009 cases with normal MB vs cases with MB abnormalities (both centres, ANOVA);

***P = 0.001 Leiden vs Genova (all cases, *χ*^2^).

^^^*P* = 0.002 cases with normal MB vs cases with MB abnormalities (Genoa, *χ*^2^).

^^^^*P*  < 0.05 cases with normal MB vs cases with MB abnormalities (Genoa, one-way ANOVA).

Three paediatric neuroradiologists with extensive experience in reporting neonatal MRIs (M.S., >10 years; M.H.L., >20 years; D.T. >6 years) and two neonatologists with long-standing experience in neonatal neurology (S.J.S., >10 years; and L.S.d.V., >30 years) independently assessed the pattern of brain injury on the MRIs with a particular focus on the limbic system, including the MBs. M.H.L., L.S.d.V., and S.J.S. assessed the MRIs in Leiden and M.S. and D.T. assessed the MRIs in Genoa.

The diagnostic criteria for the assessment of injury to the MB included:

1. Increased signal intensity with swelling of the MB over two consecutive T2-weighted slices.
2. Decreased signal intensity on T1-weighted images at the level of the MB.
3. Positive DWI findings with signal changes (high signal on trace maps and low signal on ADC maps) at the level of the MBs.

The MBs were scored as normal if no abnormal signal changes were seen on the three sequences and no swelling of the MB was noted on T2-weighted images. The MBs were considered abnormal if at least diagnostic criteria 1 was positive and, in some cases, criteria 2 and/or criteria 3 were also positive. Criteria 3 could falsely appear as negative due to the greater slice thickness or if the MRI was acquired beyond the sensitive DWI window. The criteria were the same as those used in our preliminary study^2^ and chosen to reduce the likelihood that partial volume effects were mistaken for genuine signal abnormalities given the small size and location of the MBs.

The pattern of injury, visible on the MRI, was scored using a standard, internationally recognised scoring system. Briefly, the scoring consists of four patterns: no abnormalities, damage predominantly into the watershed areas, damage predominantly to the deep grey matter (basal ganglia/thalamus), and severe brain damage, that is, near-total brain injury (Table 1). The status of the hippocampus was also assessed.

Repeat MRIs were performed in the Genoa group in a subset of 18 cases. The follow-up time for these MRIs ranged from 16 days to 6 years and 5 months.

Descriptive and comparative statistics were calculated using SPSS, version 23 (IBM, Armonk, New York).

## Results

**Fig. 1:**
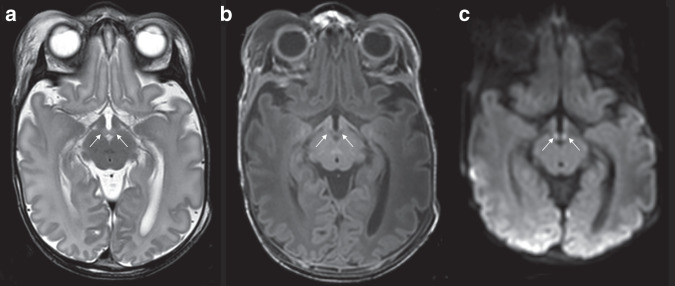
Infant with abnormal mammillary bodies. Abnormal mammillary body signal intensity and swelling on T2 (**a**) and low signal compared to normal-appearing white matter on T1 (**b**) and high signal on trace map of DWI (**c**) in a male infant, born 39 weeks GA, with perinatal asphyxia and treated with therapeutic hypothermia.

**Fig. 2:**
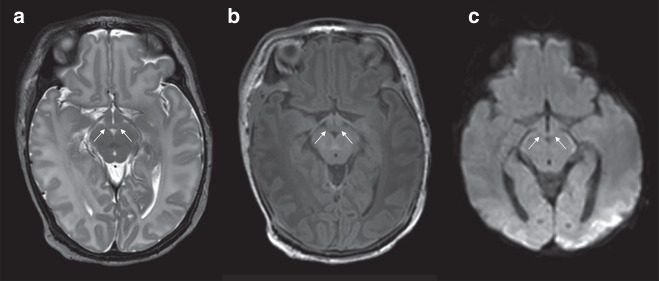
Infant with normal mammillary bodies. Normal mammillary body signal and no swelling on T2 (**a**), T1 (**b**) and DWI (**c**) in a male infant, 41 weeks GA, with perinatal asphyxia and treated with therapeutic hypothermia.

An abnormal T2 signal intensity, with swelling of the MBs, was noted in 92 of the 231 infants (41%); abnormal MB signal was observed in 36% of the cases that had been originally reported as “normal” (Figs. 1 and 2; Table 1). When abnormal MB signal was present, the brain appeared otherwise normal in 50% of the cases. When additional abnormalities were noted, the near-total brain ischaemia pattern (24%) and predominant basal ganglia/thalamus pattern (17%) were the most common, followed by the predominant watershed pattern (9%). There was, however, a significantly greater percentage of cases with near-total brain ischaemia in the group with abnormal MB signal compared to the group with normal MB signal (24 vs 8%; *p* = 0.001; see Table 1). Consistent with this, the near-total group had the highest percentage of cases with MB injury (69%; see Table 1) reflecting the widespread injury observed in this subgroup.

Abnormal MB signal was present across all grades of neonatal encephalopathy, although the most frequent occurrence was with grade II, that is, moderate, neonatal encephalopathy. However, there was very little difference when comparing encephalopathy severity in cases with and without MB involvement, suggesting that MB abnormality is not simply a result of more severe hypoxic–ischaemic episodes.

While there were more males than females in the cases identified, there was a similar occurrence of MB abnormalities across both sexes (40% of males; 45% of females; Table 2). There was some indication that abnormal MB signal was more likely to occur in infants with a slightly younger gestational age (*p* = 0.009; Table 2). For the Genoa cohort, but not the Leiden cohort, there were statistically significant differences in cord pH and Apgar score at 5 min, when comparing those cases with and without abnormal MB signal (Table 2). Likewise, in the Genoa cohort, there was a greater number of perinatal sentinel events in those cases with abnormal MB, this reflected the greater number of sentinel events in infants with the near-total pattern of damage.

The status of the hippocampus was also assessed across the two sites. For the Genoa cohort, 10 of the 83 cases had abnormal hippocampal signal and in 9 of these cases MB injury was also present. Eight of the infants with hippocampal involvement had a near-total pattern of injury and one had basal ganglia/thalamus pattern of injury. Only one case with a “normal” pattern of injury had hippocampal involvement and, in this case, the abnormal hippocampal signal was unilateral; abnormal MB signal was also present. For the Leiden cohort, 19 of the 136 cases were found to have abnormal hippocampal signal and 10 of these cases had co-occurring abnormal MB signal. Twelve of the infants with hippocampal involvement had a near-total pattern of injury and six had basal ganglia/thalamus pattern of injury. As with the Genoa cohort, only one case with a “normal” pattern of injury had abnormal hippocampal signal and, again, this co-occurred with abnormal MB signal.

In the Genoa group, of the 35 infants with abnormal MB signal, 18 patients underwent a repeat MRI. There was severe MB atrophy in 8 of these 18 patients (44%). In the Leiden group, no routine follow-up MRIs were performed.

## Discussion

We identified abnormal MB signal in neonates with encephalopathy in two centres, across two European countries, confirming the sensitivity of the MBs to perinatal hypoxia. Importantly, we demonstrated that the preliminary finding of MB involvement^2^ was not atypical, and restricted to one single centre, but likely to have a far more widespread occurrence. All of the tertiary NICUs where abnormal MB signal has been identified are dedicated centres that follow standardised care procedures in neonatal encephalopathy; they use the same criteria for initiation of hypothermia (clinical, laboratory parameters and Thompson score^9^) accepted by the international neonatology community. As such, the assumption would be that this level of MB involvement reflects the likely occurrence in all units following the same guidelines. The prevalence of abnormal MB signal was comparable across the two new sites, Leiden and Genoa (45 vs 37%), which would translate to MB pathology in a substantial number of the 20,000 live newborns with neonatal encephalopathy in Europe per year.

The present study found a normal MRI pattern in 59% (129 out of 219; Table 1) of the hypothermia-treated infants, which is consistent with this approach protecting the brain parenchyma, especially the cortex and deep grey nuclei.^3,4^ However, in 50% of cases where MB injury was present (46 out of 92; Table 1), the scans were classed as “normal” according to standard, international criteria, which is in line with our preliminary finding that hypothermia treatment provides incomplete protection against MB injury.^2^ This confirms that in the acute stage of scanning, abnormal signal can be restricted to the MBs, with other subcortical areas including the basal ganglia, thalamus and hippocampus appearing normal. This does not mean that these other structures are not affected directly by the hypoxic episode or by secondary degeneration;^10^ however, it may be that the MBs are particularly sensitive, with injury occurring earlier in the process. As such, this MB signal could act as an imaging biomarker that indicates involvement of the MBs but it also might be an early indicator of damage within the wider limbic system.

The levels of abnormal MB signal were higher than those previously reported from Utrecht^2^ where originally 18 of the 165 cases that underwent hypothermia treatment were reported to have MB pathology. However, when a subset of “borderline” scans from that centre was re-examined, a further nine were assessed as having abnormal MB signal, increasing the occurrence to 16% (27 out of 165) of cases. This has two implications, the first is that it highlights the ease in which abnormal MB signal can be missed, even when they are specifically assessed; and the second is the importance of gaining experience in examining MB signal, which makes the detection of anomalies more accurate. While the prevalence of MB abnormalities in Leiden and Genoa was still higher than in Utrecht even after this adjustment, this could reflect differences in scan protocols.

As this was a retrospective observational study, only limited follow-up scans were available. However, consistent with preliminary findings,^2^ early abnormal MB signal was shown to progress to severe MB atrophy in a number of cases where follow-up scans were carried out (8 out of 18). The MBs have repeatedly been shown to be important for memory, via their projections to the anterior thalamic nuclei.^11^ Disrupting this mammillothalamic network has widespread effects on hippocampal–cortical processing, which likely contributes to the memory impairments observed.^7^ As such, MB atrophy in these patients will likely have significant effects on episodic memory, potentially resulting in developmental amnesia.^12^ This assumption is supported by a recent paper by Cabrera-Mino et al.,^13^ where adolescents with single ventricle heart disease who had undergone the Fontan procedure showed a significant correlation between memory performance and MB volume. Patients with smaller MBs performed worse on recollective memory; this pattern mirrors that found in adults who had atrophic MBs following surgery for the removal of a colloid cyst.^14,15^ Given the similarity in cognitive findings, irrespective of whether the MB pathology was acquired in early childhood or in adulthood, it suggests that early MB pathology cannot be compensated for by other structures, reinforcing the importance of minimising injury to the MBs in neonates.

What remains unclear is the mechanism by which MB injury occurs. Interestingly, injury to the MBs does not depend on the severity of the encephalopathy. As such, it appears that a brief acute episode of severe hypoxia may be sufficient to impact the MBs, but not other structures, explaining why the brain seems otherwise normal in half of the infants with MB injury. Conversely, not all cases of near-total brain ischaemia showed abnormal MB signal in the acute phase. There are clearly situations where the MBs are protected both in the acute stage and also in those cases where abnormal signal does not result in subsequent MB atrophy. Further research, both in the clinic and using animal models, is needed to determine the conditions under which the MBs are either compromised or spared, in order to direct future treatments. One possibility might be thiamine treatment, as this is a neuroprotective agent against oxidative stress and essential for intracellular metabolism. Thiamine deficiency exacerbates damage to the MBs^16,17^ and could contribute to the pathology found following neonatal encephalopathy. While thiamine is already routinely administered to neonates with encephalopathy, it is possible that higher doses may be needed to minimise injury to the MBs.

Finally, this study highlights the importance of optimising the scanning protocols so that the MBs can be properly visualised. The T2-weighted sequence is particularly informative for assessing the status of the MBs. When 2 mm slices rather than 4 mm were used, it facilitated the detection of abnormal signal and reduced the likelihood of partial volume effects. Encouraging a more widespread practice of acquiring 2 mm T2-weighted scans in neonatal encephalopathy will enable better identification of abnormal MB signal. This is a necessary step in identifying children with a high risk of having subsequent memory problems so that they can be suitably assessed and supported.

### Limitations

This study used a multicentre, retrospective approach as this enabled us to assess whether abnormal MB signal is a feature of neonatal encephalopathy that is going undetected during routine assessment. However, there are limitations to this type of study that need to be considered when interpreting the findings. While both centres used Philips MR scanners, the scans in Leiden were carried out on a 3 T MR scanner, while in Genoa both a 1.5 and 3 T were used, with thicker slices for the T1 and T2 scans. As such, it is difficult to directly compare the rates of abnormal MB signal occurrence across sites, as the scanning protocols in Leiden could have resulted in more accurate detection of abnormal MB signal. Nevertheless, even with a less than optimal scanning protocol, which is more typically used in neonatal imaging, MB injury is observed in over a third of cases.

The MR scans were assessed by different individuals across different sites. Again, this could lead to variance and different subjective decisions as to whether an abnormal signal was present. While this makes it harder to directly compare occurrence rates across sites, it is a more realistic reflection of how abnormal MB signal would be assessed in the clinic.

The few available follow-up scans suggest that the abnormal MB signal can result in subsequent atrophy, consistent with previous findings.^2^ However, a clear goal would be to have more systematic long-term scans carried out in order to better determine which individuals are most susceptible to subsequent MB atrophy. Likewise, long-term neurodevelopmental assessments are required to evaluate how these neurological changes are reflected at the cognitive level, particularly in those cases where the scans were considered normal, and formal follow-ups were less likely to occur. We have addressed some of these issues with a follow-up of the cases from a previos cohort.^2,18^

## Conclusions

This multicentre study confirms that MB injury is commonly observed in neonatal encephalopathy. The study highlights the need for the MBs to be routinely assessed following hypoxic–ischaemic episodes and that this may require an improvement in standard scanning protocols. Abnormal MB signal is present in a substantial number of infants, despite undergoing hypothermia treatment, which could at least partly explain why infants with a history of neonatal encephalopathy have cognitive and memory deficits at school age despite having undergone therapeutic hypothermia treatment. This study reinforces the need to identify better therapeutic strategies to minimise MB injury and to carry out longitudinal assessments of those children most at risk of MB pathology.

## References

[1] Kopelman MD (1995). The Korsakoff syndrome. Br. J. Psychiatry.

[2] Molavi M, Vann SD, de Vries LS, Groenendaal F, Lequin M (2019). Signal change in the mammillary bodies after perinatal asphyxia. Am. J. Neuroradiol..

[3] Shankaran S (2009). Neonatal encephalopathy: treatment with hypothermia. J. Neurotrauma.

[4] Rutherford M (2010). Assessment of brain tissue injury after moderate hypothermia in neonates with hypoxic-ischaemic encephalopathy: a nested substudy of a randomised controlled trial. Lancet Neurol..

[5] Lee-Kelland R (2020). School-age outcomes of children without cerebral palsy cooled for neonatal hypoxic-ischaemic encephalopathy in 2008–2010. Arch. Dis. Child Fetal Neonatal Ed..

[6] Campbell H (2018). Hypothermia for perinatal asphyxia: trial-based quality of life at 6–7 years. Arch. Dis. Child..

[7] Dillingham CM (2019). Mammillothalamic disconnection alters hippocampocortical oscillatory activity and microstructure: Implications for diencephalic amnesia. J. Neurosci..

[8] Zheng Q, Viaene AN, Freeman CW, Hwang M (2020). Radiologic-pathologic evidence of brain injury: hypoperfusion in the Papez circuit results in poor neurodevelopmental outcomes in neonatal hypoxic ischemic encephalopathy. Childs Nerv. Syst..

[9] Thompson CM (1997). The value of a scoring system for hypoxic ischaemic encephalopathy in predicting neurodevelopmental outcome. Acta Paediatr..

[10] Williams CE, Gunn AJ, Mallard C, Gluckman PD (1992). Outcome after ischemia in the developing sheep brain: an electroencephalographic and histological study. Ann. Neurol..

[11] Dillingham CM, Frizzati A, Nelson AJ, Vann SD (2015). How do mammillary body inputs contribute to anterior thalamic function?. Neurosci. Biobehav. Rev..

[12] Dzieciol AM (2017). Hippocampal and diencephalic pathology in developmental amnesia. Cortex.

[13] Cabrera-Mino C (2020). Reduced brain mammillary body volumes and memory deficits in adolescents who have undergone the Fontan procedure. Pediatr. Res..

[14] Vann SD (2009). Impaired recollection but spared familiarity in patients with extended hippocampal system damage revealed by 3 convergent methods. Proc. Natl Acad. Sci. USA.

[15] Tsivilis D (2008). A disproportionate role for the fornix and mammillary bodies in recall versus recognition memory. Nat. Neurosci..

[16] Kornreich L (2005). Thiamine deficiency in infants: MR findings in the brain. Am. J. Neuroradiol..

[17] Zuccoli G, Siddiqui N, Bailey A, Bartoletti SC (2010). Neuroimaging findings in pediatric Wernicke encephalopathy: a review. Neuroradiology.

[18] Annink, K. et al. Mammillary body atrophy and other MRI correlates of school-age outcome following neonatal hypoxic-ischemic encephalopathy. Scientific Reports. (in press). 10.1038/s41598-021-83982-8 PMC7930036 33658541

